# spliceR: an R package for classification of alternative splicing and prediction of coding potential from RNA-seq data

**DOI:** 10.1186/1471-2105-15-81

**Published:** 2014-03-23

**Authors:** Kristoffer Vitting-Seerup, Bo Torben Porse, Albin Sandelin, Johannes Waage

**Affiliations:** 1 Department of Biology, The Bioinformatics Centre, University of Copenhagen, Ole Maaloes Vej 5, Copenhagen, DK2200, Denmark; 2 Biotech Research and Innovation Centre (BRIC), University of Copenhagen, Ole Maaloes Vej 5, Copenhagen, DK-2200, Denmark; 3 The Finsen Laboratory, Rigshospitalet, Faculty of Health Sciences, University of Copenhagen, Ole Maaloes Vej 5, Copenhagen, DK2200, Denmark; 4 The Danish Stem Cell Centre (DanStem) Faculty of Health Sciences, University of Copenhagen, Ole Maaloes Vej 5, Copenhagen, DK2200, Denmark

**Keywords:** spliceR, RNA-Seq, Alternative splicing, Nonsense mediated decay (NMD), Isoform switch

## Abstract

**Background:**

RNA-seq data is currently underutilized, in part because it is difficult to predict the functional impact of alternate transcription events. Recent software improvements in full-length transcript deconvolution prompted us to develop spliceR, an R package for classification of alternative splicing and prediction of coding potential.

**Results:**

spliceR uses the full-length transcript output from RNA-seq assemblers to detect single or multiple exon skipping, alternative donor and acceptor sites, intron retention, alternative first or last exon usage, and mutually exclusive exon events. For each of these events spliceR also annotates the genomic coordinates of the differentially spliced elements, facilitating downstream sequence analysis. For each transcript isoform fraction values are calculated to identify transcript switching between conditions. Lastly, spliceR predicts the coding potential, as well as the potential nonsense mediated decay (NMD) sensitivity of each transcript.

**Conclusions:**

spliceR is an easy-to-use tool that extends the usability of RNA-seq and assembly technologies by allowing greater depth of annotation of RNA-seq data. spliceR is implemented as an R package and is freely available from the Bioconductor repository ( http://www.bioconductor.org/packages/2.13/bioc/html/spliceR.html).

## Background

Alternative splicing is one of the most important RNA modifications, leading to protein diversification and contributing to the complexity of higher organisms [1]. Recent advances in RNA sequencing (RNA-seq), combined with modern RNA-seq assembly software such as Cufflinks [2], allows for high-resolution profiling of the RNA landscape. The technological and computational advances enables identification of a catalog of distinctly spliced transcripts originating from the same transcription unit/gene. These full-length transcripts are however underutilized, in part because it is difficult to predict the functional impact of alternate transcription events leading to the different transcripts. In addition to the potential for impact on protein domains, alternate splicing may also alter RNA processing, stability and localization. Nonsense mediated decay (NMD) is tightly linked to alternate splicing, and the mechanism by which even small changes in alternative splicing can result in degradation via NMD is well described [3]. To predict splicing events that may lead to these functional changes, it is necessary to classify the type of event as well as annotate the genomic position of the regions that are differentially spliced. Such classifications also enable systematic follow-up analyses, such as sequence analysis of the differentially spliced regions to infer the underlying mechanisms. However, at present there are no available tools that adequately perform these analyses. Existing methods, including MISO [4], Astalavista [5] and DiffSplice [6], do not output the genomic coordinates of differentially spliced regions [4,7-11], have insufficient classification of alternative splicing (i.e., only a subset of alternative splicing classes are supported) [4-11], or cannot assess novel features [4,7,8,12]. Furthermore, none of the existing tools for analyzing alternative splicing support NMD predictions [4-12].

This prompted us to develop the R package spliceR. spliceR uses the full-length transcripts created by RNA-seq assemblers to detect single- and multiple exon skipping/inclusion (ESI, MESI), alternative donor and acceptor sites (A5, A3), intron retention (IR), alternative first or last exon usage (ATSS, ATTS), and mutually exclusive exon events (MEE). spliceR annotates the genomic coordinates of the differentially spliced elements, facilitating downstream sequence analysis. Finally, spliceR predicts the coding potential of transcripts, calculates untranslated region (UTR) and open reading frame (ORF) lengths and predicts whether transcripts are NMD-sensitive based on compatible annotated start codon positions and their downstream ORF.

## Implementation

### Retrieval of data

spliceR is implemented as an R package and is freely available from the Bioconductor repository ( http://www.bioconductor.org/packages/2.13/bioc/html/spliceR.html). It is based on standard Bioconductor [13] classes such as GRanges, allowing for full flexibility and modularity, and support for all species and versions supported in the Bioconductor annotation packages. An example dataset is included to allow easy exploration of the package.

**Figure 1:**
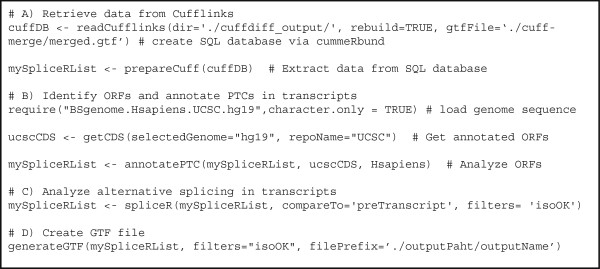
**The R code for a standard spliceR analysis.** The R code necessary to run a standard spliceR analysis based on Cuffdiff output. **A)** The R code to generate a spliceRList from Cuffdiff output. **B-D)** The R code for making a standard spliceR analysis.

spliceR is compatible with the output from any full-length RNA-seq assembler, but was designed to integrate with Cufflinks and includes a dedicated function that retrieves all relevant information from the SQL database generated by Cufflinks axillary R package cummeRbund. In future versions new functions dedicated to import data from other RNA-seq assemblers will be introduced. The R code for a standard workflow based on Cufflinks data is illustrated in Figure 1. A workflow using output from other full-length RNA-seq assemblers is provided in the spliceR Bioconductor documentation.

### Classification of alternative splicing

**Figure 2:**
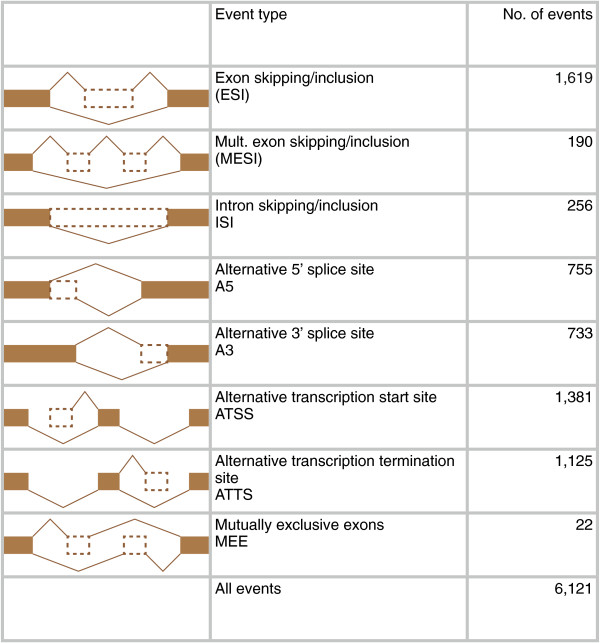
**Number of individual alternative splicing events identified.** A schematic structure of each alternative splicing type, along with the associated name, abbreviation and the number of classified events in Usp49 KD RNA seq data.

For each gene, spliceR constructs the hypothetical pre-RNA based on the exon information from all transcripts originating from that gene. Subsequently, all transcripts are compared to this hypothetical pre-RNA in a pairwise manner, and alternative splicing events are classified and annotated (see Figure 2 for a schematic overview). Alternatively, spliceR can be configured to use the most expressed transcript as the reference transcript instead of the theoretical pre-RNA. This may be useful in perturbation scenarios where investigators are interested in deviation from normal conditions. For statistical assessment of differential splicing, users can access the transcript fidelity status and *P*-values of Cufflinks, or can easily apply other R-packages that are tailored for this purpose, including edgeR [14], deseq [15], and baySeq [16].

### Isoform fraction values

For each transcript and condition, spliceR calculates an isoform fraction (IF) value, which is calculated as (transcript expression) / (gene expression) *100 to represent the contribution of a transcript to the expression of the parent gene. Furthermore delta-IF (dIF) values, which measure the absolute change in IF values between conditions, are also calculated. Since these dIF values measure changes in relative abundance of isoforms within a gene, they are well suited for identifying and analyzing changes in the isoform usage. Such analysis does however require that the expression values of transcripts and genes have been normalized to length, sequencing depths, and possibly other biases, a standard feature of the Cufflinks FPKM (fragments per kilobase of transcript per million fragments sequenced) metric. For data from other assemblers, the user may need to accommodate this requirement manually. These IF values are highly dependent on the underlying data quality and relative expression strengths, and should be viewed as a helpful ranking statistic for identifying changes in alternative splicing between conditions.

### Analysis of coding potential

spliceR retrieves the genomic exon sequences of each transcript from one of the Bioconductor annotation files. ORF annotation is then retrieved from the UCSC Genome Browser repository either from RefSeq, UCSC or Ensembl, as specified by the user. Alternatively, a custom ORF-table can be supplied. For each transcript, the most upstream compatible start codon is identified, the downstream sequence is translated, and the relative position of the first in-frame stop codon and the distance to the final exon-exon junction is recorded and returned to the user. Transcripts are marked NMD-sensitive if the stop codon falls more than 50 nt upstream of the final exon-exon junction, indicating a pre-mature stop codon (PTC). This is based on the literature consensus [17], however this setting is user-configurable. The position of the start codon is also annotated, which, in combination with the found stop codon and the annotated transcript lengths, enables users to calculate 5′UTR lengths, ORF lengths and 3′UTR lengths. In future versions, we plan to include alternative methods of coding region prediction, such as the logistic regression model implemented in the program CPAT [18].

### Visualization and data integration tools

The spliceR package generates a GTF file that can be uploaded to the UCSC genome browser, to help users visualize transcripts and to allow for integration of the RNA-seq analysis with external annotation sources. This spliceR GTF file has two main advantages over the corresponding GTF file generated by Cufflinks’ Cuffmerge tool: Firstly, spliceR’s optional filters uses stringent criteria, e.g. requiring that the transcript should be expressed, or that Cufflinks marks the transcript deconvolution as successful. In our experience, this removes up to 80% of transcripts originally predicted to belong to the same gene in the Cufflinks GTF file (data not shown), making the GTF file generated by spliceR more suitable for visual analysis.

Secondly, spliceR can color-code transcripts according to their expression level within the parent gene. This feature facilitates easy visualization of changes in gene and transcript expression both within and between conditions.

The tabulated output of spliceR facilitates a number of downstream analyses, including identification of transcripts that exhibit major changes between samples, the ability to filter the output for specific splicing classes, as well as sequence analysis in or around regions that are spliced in or out between samples. Examples include detection of enriched motifs, or identification of protein domains, miRNA response elements or localization signals that are spliced in or out. spliceR facilitates these types of analyses by outputting the genomic coordinates of each alternatively spliced element.

## Results and discussion

To show the power and versatility of spliceR we reanalyzed the publically available RNA-seq dataset from Zhang *et al.*[19], which compared two experimental conditions: the human colorectal cell line HT116 with and without a siRNA directed towards Usp49, a histone H2B deubiquitinase. To compare our approach with the original analysis, we used the original Cufflinks data (GEO: GSE38100). Transcripts successfully deconvoluted by cufflinks were used as the input into spliceR. The resulting dataset contained 5,496 single-transcript genes, and 1,867 multi-transcript genes. Combined, the multi-transcripts genes were predicted to express 4,612 unique transcripts 2.47 transcripts per gene). The analysis and results presented here are based on only five lines of R code, (modified to hg18), shown in Figure 1B-D, which illustrates the power and ease-of-use of spliceR. For reference, the spliceR analysis took ~30 minutes on a typical laptop (MacBook Pro 2.5Ghz i5, 8 GB RAM).

### Splicing pattern and transcript structure

To validate the transcripts generated by Cufflinks, the two first and two last nucleotides of all introns, corresponding to the locations of splice site consensus sequences, were extracted from both the transcripts generated by Cufflinks as well as reference transcripts from RefSeq and Gencode. The extracted dinucleotides were then compared to the canonical splicing motifs and the percentage of dinucleotides agreeing with the classical splice site motif was analyzed (Table 1). The transcripts predicted by Cufflinks were spliced in accordance with the hyper-conserved splicing motifs as frequently as transcripts originating from RefSeq or Gencode. To further validate the transcripts obtained though Cufflinks we used Cufflinks’ cuffcompare tool (v 2.1.1) against all RefSeq transcripts. This showed that the sensitivity and specificity for both introns and exons detection are high (always over 0.91 and 0.81, respectively, and typically much higher) (Table 2). These two analyses indicate that that the splicing pattern and transcript structure observed in the Cufflinks derived transcripts are of high quality and suitable for further analyses.

**Table 1:** Frequency of splice site consensus sequences

	**Usp49 RNA-seq**	**Gencode**	**Refseq**
5′ end (GT)	93.23	89.72	92.08
3′ end (AG)	93.70	90.64	92.71

The two first and last nucleotides, corresponding to the splice site consensus sequences, were extracted from all exons originating from the RNA-seq data, Gencode, and RefSeq. The percentage of dinucleotides identical to the canonical motif was calculated.

**Table 2:** Comparison of the analyzed transcripts to RefSeq

	**WT GTF**		**Usp49KD GTF**	
	**Sensitivity**	**Specificity**	**Sensitivity**	**Specificity**
Exon level	97.90	89.20	98.00	88.70
Intron level	96.40	97.00	96.40	96.90
Intron chain level	91.70	81.90	91.80	81.90

Cufflinks’ cuffcompare tool (v. 2.1.1.) was used to compare RefSeq to the obtained transcripts and the sensitivity and specificity (as defined [20]) for exons, introns, and intron chains are shown.

### Alternative splicing and NMD

From the Usp49 KD dataset spliceR identified a total of 6,121 alternative splicing events (Additional file 1: Table S1), distributed across the different splicing classes shown in Figure 2. spliceR found 8,179 (80.9%) transcripts without a PTC (PTC-), 642 (6,4%) transcripts with a PTC (PTC+) while 1,287 (12.7%) transcripts did not have any annotated compatible start codons. Similar fractions of transcripts were predicted to be NMD sensitive when all transcripts from RefSeq (8.2%) and Gencode (9.7%) were analyzed with spliceR, indicating that a non-neglectable fraction of transcripts could be NMD sensitive. By using spliceR’s annotation of start and stop codons, the length of both 5′UTRs, 3′UTRs and ORFs were analyzed, but no changes between conditions were found (data not shown).

### Splicing efficiency

**Figure 3:**
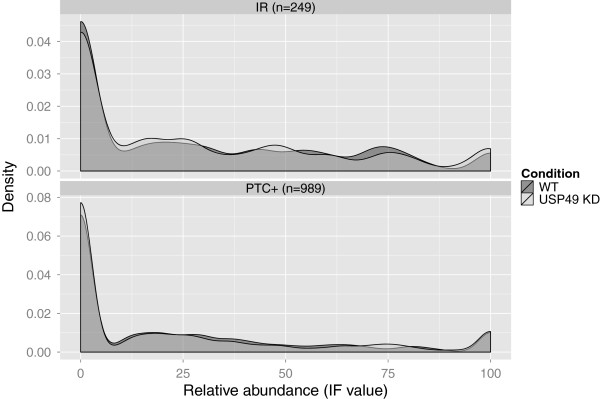
**Relative abundance of transcripts.** All NMD + transcripts (bottom) and all transcripts with IR (top) was extracted and the density distributions of the IF values from WT and Usp49 KD were plotted.

Zhang and colleagues reported that a subset of transcripts were enriched for intron retention following Usp49 depletion [19], leading to the hypothesis that Usp49 KD reduced the splicing efficiency of pre-RNA molecules. If Usp49 KD impaired splicing efficiency, an increase in the relative abundance of transcripts with IR when comparing WT and KD would be expected. Since the relative abundance of transcripts is measured by IF values, we tested this hypothesis by comparing the distributions of IF values from the subset of transcripts with IR (Figure 3). This analysis shows that transcripts with IR had no global changes in their relative abundance (*P* = 0.71, Mann–Whitney U test) indicating that the global splicing efficiency was unchanged. This type of analysis could however be used to analyze changes in isoform usage in any subset of transcripts that the user could find interesting, for example all NMD sensitive transcripts (Figure 3).

### Transcript switching

We next assessed transcripts whose relative abundance was altered by the Usp49KD, by filtering for genes that had both a large positive and large negative dIF value (corresponding to a binary transcript-switch). 183 high confidence transcript switches were found: in 18 instances (~9.8%), an NMD-negative transcript was down-regulated while a NMD-sensitive transcript was up-regulated. This illustrates that failing to assess the NMD sensitivity can lead to overestimation of the number of functionally relevant transcript switches.

**Figure 4:**
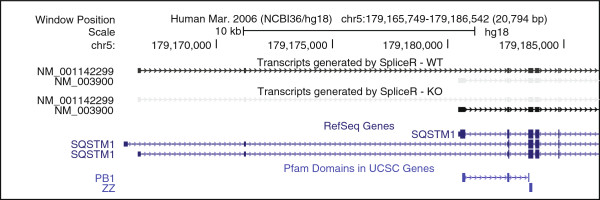
**Example of transcript switching.** Screen shot from the UCSC genome browser showing the transcript switch found in the SQSTM1 gene. The two top tracks show transcripts generated by the generateGTF() function for WT (top) and Usp49KD (bottom). Darker transcripts are expressed at higher levels. The two bottom tracks indicate RefSeq genes (top) and protein domains identified via Pfam [21] respectively (bottom).

The transcript switch in the SQSTM1 gene (Figure 4) illustrates the utility of integrating the spliceR data with information in the UCSC genome browser to identify functional changes conferred by alternate splicing. Visual inspection of the isoform switch was possible by uploading the GTF file generated by spliceR. As seen in Figure 4, KD of Usp49 caused a switch from the long transcript predicted to contain a truncated PB1 domain, to the short transcript predicted to encode an intact PB1 domain.

## Conclusion

Here, we have introduced the R package spliceR, which increases the usability and power of RNA-seq and assembly technologies by providing a full overview of alternative splicing events and protein coding potential of transcripts. spliceR is flexible and easily integrated in existing workflows, supports input and output of standard Bioconductor data types, and enables investigators to perform many different downstream analyses of both transcript abundance and differentially spliced elements. We demonstrate the power and versatility of spliceR by showing how new conclusions can be made from existing RNA-seq data.

## Availability and requirements

SpliceR is implemented as an R package, is freely available from the Bioconductor repository and can be installed simply by copy/pasting two lines into an R console.

•**Project nam**e: spliceR

•**Project home page:**http://www.bioconductor.org/packages/2.13/bioc/html/spliceR.html

•**Operating system(s):** Platform independent

•**Programming language:** R and C

•**Other requirements:** R v 3.0.2 or higher

•**License:** GPL

•**Any restrictions to use by non-academics:** No limitations

## Competing interests

The authors declare that they have no competing interests.

## Authors’ contributions

KVS and JW developed the R package. BP, AS, KVS and JW planned the development and wrote the article. All authors read and approved the final manuscript.

## Supplementary Material

Additional file 1: Table S1 Tabulated output of the spliceR analysis. Click here for file
